# Psychometric properties of patient-reported outcome measures for health-related quality of life in multiple myeloma: a systematic review based on COSMIN guidelines

**DOI:** 10.1186/s12885-026-16341-3

**Published:** 2026-06-15

**Authors:** Lili Xie, Qiong Feng, Jiaxin Wang, Weiyi Qin, Yuhong Ding, Zhishui Wu

**Affiliations:** 1 School of Nursing and Rehabilitation, Nantong University, Nantong, Jiangsu Province China; 2 Department of Hematology, Nantong University Affiliated Hospital, Nantong, Jiangsu Province China; 3 Department of Spine Surgery, The First People’s Hospital of Changzhou, Changzhou, Jiangsu Province China

**Keywords:** Multiple Myeloma, Patient-Reported Outcome Measures, Psychometric Properties, Health-Related Quality of Life, Systematic Review, COSMIN

## Abstract

**Purpose:**

To systematically identify patient-reported outcome measures (PROMs) used to assess health-related quality of life (HRQoL), symptoms, and disease burden in patients with multiple myeloma (MM), and to appraise their measurement properties using the COSMIN methodology.

**Methods:**

A systematic review was conducted in accordance with the COSMIN guideline for reviews of PROMs. Eligible studies reported PROM development, content validation, cross-cultural adaptation, or one or more measurement properties of PROMs used in adult patients with MM. Methodological quality was assessed using the COSMIN Risk of Bias checklist. The results for each measurement property were rated against the COSMIN criteria for good measurement properties, and the certainty of evidence was graded using the modified GRADE approach recommended by COSMIN.

**Results:**

Twenty-six studies involving 14 PROMs were included. The EORTC QLQ-MY20 was the most frequently evaluated MM-specific module, followed by MyPOS, HM-PRO, MDASI-MM, FACT-MM, MySIm-Q, and QLICP-MM. Evidence was most often available for internal consistency, structural validity, and hypothesis testing for construct validity. By contrast, formal evidence on measurement error, criterion validity, and cross-cultural validity was seldom reported. No PROM demonstrated uniformly high-quality evidence across all key measurement properties. The EORTC QLQ-MY20 and MyPOS showed the broadest body of supportive evidence and appear to be the most defensible options for MM-specific HRQoL assessment, although additional high-quality content validity and longitudinal measurement studies remain necessary.

**Conclusion:**

Current evidence supports the use of several MM-specific PROMs, particularly the EORTC QLQ-MY20 and MyPOS, but the overall evidence base remains uneven. Future research should prioritize patient-centered content validation, measurement-error evidence, cross-cultural validity, responsiveness, and interpretability to strengthen PROM selection for MM clinical trials and practice.

**Supplementary Information:**

The online version contains supplementary material available at 10.1186/s12885-026-16341-3.

## Plain Language Summary

Multiple myeloma patients often experience pain, fatigue, and other issues affecting their quality of life during treatment. To accurately understand patients’ experiences, doctors use various questionnaires (instruments). This study systematically reviewed all currently used instruments for multiple myeloma, analyzing their scientific rigor and accuracy. QLQ-MY20 and MyPOS have shown good psychometric properties in current evidence, but they have missing or low-quality evidence for some measurement attributes. No single PROM has high-quality evidence for all attributes. However, most instruments require further validation for cross-cultural use (e.g., in different countries). This research helps doctors choose the most appropriate instruments for monitoring patient health.

**Supplementary Information:**

The online version contains supplementary material available at 10.1186/s12885-026-16341-3.

## Introduction

Multiple Myeloma (MM) is the second most common hematologic malignancy, characterized by the clonal proliferation of plasma cells in the bone marrow [1]. With the widespread application of novel therapies such as proteasome inhibitors, immunomodulatory drugs, monoclonal antibodies, and chimeric antigen receptor T-cell (CAR-T) therapy, the survival of MM patients has significantly improved, transforming the disease pattern into a chronic condition requiring long-term management [2, 3]. However, the disease itself and its treatment are often accompanied by a constellation of symptoms, including bone pain, anorexia, fatigue, increased infection risk, renal impairment, and peripheral neuropathy. These factors collectively exert a profound impact on patients’ physical function, psychological state, and social roles, leading to a significant decline in Health-Related Quality of Life (HRQoL) [4, 5]. Therefore, a comprehensive and accurate assessment of HRQoL in MM patients is of critical clinical importance for optimizing individualized treatment decisions, evaluating the net benefit of therapeutic regimens, and improving the long-term survivorship experience.

Patient-Reported Outcomes (PROMs) are reports coming directly from patients about their health status and treatment, without interpretation by healthcare professionals or others [6]. In MM clinical practice and research, PROMs have become central instruments for assessing symptom burden, treatment toxicity, and HRQoL. They can capture patients’ subjective experiences that are difficult to reflect through clinical examination or laboratory parameters, facilitate doctor-patient communication, enable early identification of unmet needs, and ultimately enhance the quality of patient-centered care [7, 8]. Professional bodies such as the International Myeloma Working Group (IMWG) and the European Society for Medical Oncology (ESMO) recommend integrating PROMs into routine MM management and clinical trials [9, 10].

Currently, a variety of PROMs are used to assess quality of life in MM patients, primarily falling into two categories: generic and disease-specific instruments. Generic HRQoL instruments, applicable to the general population, cancer patients, or those with hematologic malignancies, include the Medical Outcome Study Short Form 36 (SF-36) [11], the Quality of Life Questionnaire Core 30 (QLQ-C30) [12], the EQ-5D-5 L [13], and the Hematological Malignancy Patient-Reported Outcome measure (HM-PRO) [14]. Disease-specific instruments are designed specifically for MM patients, such as the European Organisation for Research and Treatment of Cancer Quality of Life Questionnaire Myeloma Module (EORTC QLQ-MY20) [15] and the Functional Assessment of Cancer Therapy – Multiple Myeloma (FACT-MM) [16], the Multiple Myeloma Symptom and Impact Questionnaire (MySIm-Q) [17], the Quality of Life Instruments for Cancer Patients with Multiple Myeloma (QLICP-MM) [18], the Myeloma Patient Outcome Scale (MyPOS) [19], and the M.D. Anderson Symptom Inventory – Multiple Myeloma Module (MDASI-MM) [20]. Additionally, several cancer symptom-focused instruments, like the Functional Assessment of Cancer Therapy – General (FACT-G) [21], the Memorial Symptom Assessment Scale (MSAS) [22], its Short Form (MSAS-SF) [23], and the Edmonton Symptom Assessment System (ESAS) [24], are also frequently employed in the MM population.

Despite the availability of numerous tools, the psychometric properties of these instruments in the MM patient population have not been systematically summarized and compared. The methodological quality of instrument development and validation processes varies across studies, posing challenges for clinicians and researchers in selecting the most appropriate and robust measurement instruments. The Consensus-based Standards for the selection of health Measurement Instruments (COSMIN) initiative provides a rigorous methodological framework for systematically reviewing the measurement properties of PROMs and the methodological quality of their studies, offering evidence-based graded recommendations for instrument selection [25, 26].

In this context, and to provide clear guidance for clinical practice and research, there is an urgent need to comprehensively evaluate existing HRQoL instruments used in MM patients employing the COSMIN methodology. This systematic review aims to: (1) systematically identify and evaluate PROMs developed or validated for MM patients; (2) critically appraise the methodological quality of evidence for these instruments’ various psychometric properties (e.g., content validity, structural validity, reliability, responsiveness); (3) synthesize the body of evidence to provide clear, evidence-based recommendations for instrument selection under different measurement purposes. Through this work, we aim to promote the standardization and scientific rigor of HRQoL assessment in MM, ultimately benefiting patient care and clinical research.

## Methods

### Design

This systematic review was conducted according to the COSMIN methodology for systematic reviews of PROMs. The review focused on PROMs that had been developed, adapted, translated, or psychometrically evaluated for use in patients with MM [25, 27]. Throughout the manuscript, the term “PROM” or “instrument” is used rather than “scale”, unless a specific instrument is formally named as an instrument. The study protocol was registered prospectively in the International Prospective Register of Systematic Reviews (PROSPERO) (Registration number: CRD420251127878). Reporting will follow the Preferred Reporting Items for Systematic reviews and Meta-Analyses (PRISMA) – COSMIN Extension statement [28].

### Search strategy

A systematic search was performed across eight databases: PubMed, Web of Science, Embase, CINAHL, Cochrane Library, SinoMed, China National Knowledge Infrastructure, and Wanfang Data. The search covered studies published from the inception of each database until August 26, 2025. References of included studies were also tracked. The search strategy combined Medical Subject Headings (MeSH) terms, keywords, and search filters [29] to identify studies related to measurement properties. Detailed search strategies for all databases are provided in Supplementary S3.

### Eligibility criteria

Studies were eligible if they reported the development, content validation, cross-cultural adaptation, or evaluation of at least one measurement property of a PROM used to assess HRQoL, symptoms, functioning, disease burden, or treatment-related impact in adults with MM. Eligible instruments included MM-specific PROMs and broader cancer-, hematology-, or generic HRQoL instruments when they were evaluated in an MM population or were commonly used as comparator or companion measures in MM studies. Studies were excluded if they were not published in English or Chinese; were secondary literature, such as reviews, systematic reviews, meta-analyses, conference abstracts, commentaries, or books; had no available full text; reported duplicate data; or reported only clinical outcomes without PROM measurement-property evidence.

### Study selection

Two reviewers familiar with the COSMIN guidelines and evidence-based medicine independently performed the literature screening. First, duplicate records were removed using the reference management software EndNote X20. Subsequently, titles and abstracts were screened to exclude obviously irrelevant studies. The full texts of the remaining records were read to determine final inclusion. If no validation study in MM patients was retrieved for a specific instrument, its original publication or landmark validation study was traced and included to assess the instruments’ basic psychometric properties. Disagreements during screening were resolved through discussion with a third senior methodological researcher. The entire screening process and reasons for exclusion were documented.

### Data extraction and analysis

For each included study, two reviewers independently extracted publication details, country and language, PROM name, target construct, target population, sample size, item number, response options, recall period, scoring method, and all reported measurement properties. Extracted measurement properties were mapped to the COSMIN taxonomy: PROM development, content validity, structural validity, internal consistency, cross-cultural validity/measurement invariance, reliability, measurement error, criterion validity, hypothesis testing for construct validity, and responsiveness.

### Methodological quality assessment

Methodological quality was evaluated with the COSMIN Risk of Bias checklist [27]. Each study was assessed separately for each measurement property that it reported. The PROM development box and the content validity box were judged independently, because they address different evidence sources: the adequacy of the instrument-development process and the quality of studies evaluating relevance, comprehensiveness, and comprehensibility. Each box was rated as very good, adequate, doubtful, or inadequate using the COSMIN “worst score counts” principle.

### Evaluation of psychometric property

The results for each measurement property were rated as sufficient (+), insufficient (−), or indeterminate (?) using the COSMIN criteria for good measurement properties [27, 30, 31]. For example, internal consistency was considered sufficient when Cronbach’s alpha was ≥ 0.70 and structural validity for the corresponding instrument or subscale was at least supported [27]. Reliability was considered sufficient when the intraclass correlation coefficient or weighted kappa was ≥ 0.70 [27]. Hypothesis testing for construct validity and responsiveness were considered sufficient when at least 75% of predefined or review-team hypotheses were confirmed [27]. Content validity was summarized across relevance, comprehensiveness, and comprehensibility, while distinguishing evidence from PROM-development studies from evidence from additional content-validity studies [27].

### Evidence synthesis and grading

For each PROM and measurement property, the certainty of evidence was graded as high, moderate, low, or very low using the modified GRADE approach recommended by COSMIN, taking into account methodological quality, inconsistency, imprecision, and indirectness [25, 27]. Final recommendations were categorized as follows [25, 27]: category A, PROMs with evidence for sufficient content validity and at least low-quality evidence for sufficient internal consistency; category B, PROMs with potential for use but requiring further validation; and category C, PROMs with high-quality evidence for an insufficient measurement property and therefore not recommended for the target context.

## Results

### Study selection

**Fig. 1:**
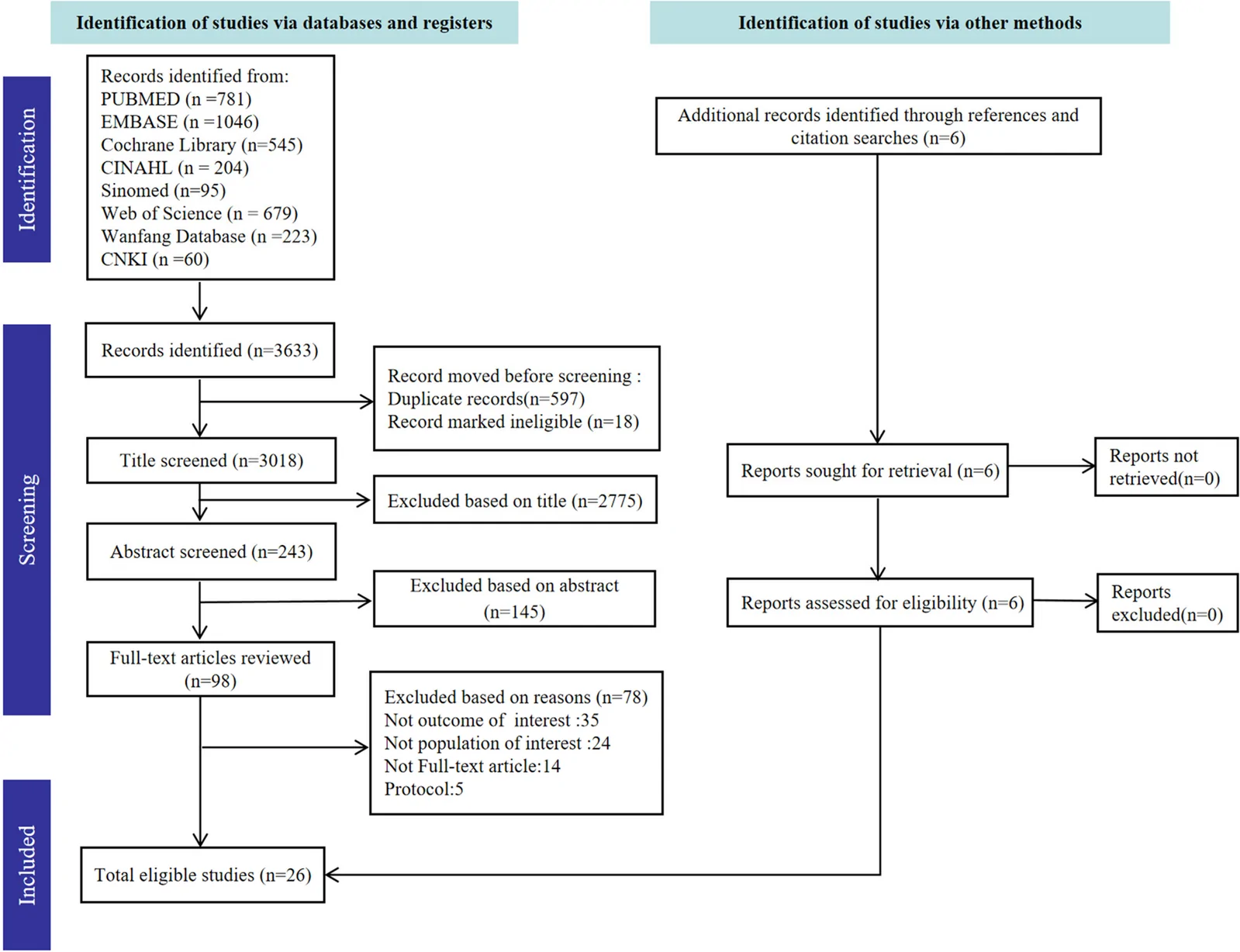
PRISMA flow diagram illustrating screening and selection of articles

A total of 3633 records were initially identified (Fig. 1). After removing 597 duplicates and 18 ineligible records, 3018 records were screened. Title screening excluded 2775 records. Abstract review of 243 records led to the exclusion of 145. Full-text assessment of 98 articles resulted in the selection of 20 articles based on eligibility criteria. An additional 6 articles were identified through manual searches, culminating in 26 articles [18, 32–56] included in the systematic review of quality of life in multiple myeloma patients.

### Study characteristics

After screening, 26 studies were included in the review. The included studies covered 14 PROMs relevant to MM, including MM-specific measures, hematological malignancy measures, cancer-specific measures, symptom measures, and generic HRQoL instruments. The most frequently studied MM-specific instrument was the EORTC QLQ-MY20, which had been evaluated in multiple languages and cultural contexts. Other MM-relevant instruments included MyPOS, FACT-MM, MDASI-MM, MySIm-Q, QLICP-MM, and HM-PRO. Generic or broader oncology instruments included the EORTC QLQ-C30, FACT-G, MSAS, MSAS-SF, ESAS, EQ-5D-5 L, and SF-36. Study characteristics are summarized in Table 1.

**Table 1:** Characteristics of included studies

PROM	Year	Study	Country/language	Purpose	Sample/target population	Measurement properties addressed
EORTC QLQ-MY20	2007	K Cocks [32]	International/English	Development and field testing of an MM module	Adults with MM; international field-test sample	PROM development, content validity, structural validity, internal consistency, construct validity, responsiveness
EORTC QLQ-MY20	2012	Kontodimopoulos, N. [33]	Greece/Greek	Translation and validation	Greek patients with MM	Structural validity, internal consistency, reliability, construct validity
EORTC QLQ-MY20	2015	Espinoza-Zamora, J. R [34]	Mexico/Spanish	Cultural adaptation and validation	Mexican patients with MM	Internal consistency, test–retest reliability, construct validity, responsiveness
EORTC QLQ-MY20	2016	Ahmadzadeh, A. [35]	Iran/Persian	Translation and validation	Persian-speaking patients with MM	Internal consistency, reliability, construct validity
EORTC QLQ-MY20	2023	Dachs, L. R. [36]	Brazil/Portuguese	Validation in Brazilian Portuguese	Brazilian patients with MM	Internal consistency; hypothesis testing for construct validity, including known-groups comparisons
EORTC QLQ-MY20	2024	Lorca, L. A. [37]	Chile/Spanish	Validation in Chilean Spanish	Chilean patients with MM	Internal consistency, construct validity
EORTC QLQ-MY20	2025	Chao Liu [38]	China/Chinese	Translation and psychometric validation	Chinese patients with MM	Content validity, structural validity, internal consistency, reliability, construct validity
MyPOS	2015	Osborne, T. R [39]	United Kingdom/English	Development and validation	Patients with MM across clinical settings	PROM development, content validity, structural validity, internal consistency, reliability, construct validity
MyPOS	2017	Ramsenthaler, C. [40]	United Kingdom/English	Clinical validation and responsiveness testing	Patients with MM receiving care	Structural validity, internal consistency, reliability, construct validity, responsiveness
MyPOS	2020	Gerlach, C. [41]	Germany/German	Translation and cultural adaptation	German-speaking patients with MM	Cross-cultural adaptation, content validity, feasibility
FACT-MM	2012	Wagner, L.I. [42]	United States/English	Development of an MM-specific FACT module	Patients with MM and clinicians	PROM development, content validity, internal consistency, construct validity
MDASI-MM	2013	Jones, D. [43]	United States/English	Development and validation of an MM symptom module	Patients with MM undergoing therapy or transplantation	PROM development, structural validity, internal consistency, reliability, construct validity
MDASI-MM	2025	Ting Wang [44]	China/Chinese	Translation and validation	Chinese patients with MM	Content validity, structural validity, internal consistency, reliability, construct validity
MySIm-Q	2021	Gries, K. S. [45]	International/English	Development and psychometric validation	Patients with MM	PROM development, content validity, structural validity, internal consistency, reliability, construct validity
QLICP-MM	2022	Li Weiqang [18]	China/Chinese	Development and validation of an MM module	Chinese patients with MM	PROM development, structural validity, internal consistency, reliability, construct validity, responsiveness
HM-PRO	2020	Goswami, P. [46]	United Kingdom/English	Content validation	Patients with hematological malignancies including MM	PROM development, content validity
HM-PRO	2020	Goswami, P. [47]	United Kingdom/English	Psychometric validation	Patients with hematological malignancies including MM	Structural validity, internal consistency, reliability, construct validity
HM-PRO	2025	Sam Salek [48]	International/multilingual	Further validation and use in hematologic malignancies	Patients with hematological malignancies including MM	Content validity, construct validity, feasibility
EORTC QLQ-C30	1993	Ringdal, G. I. [49]	Norway/Norwegian	Cancer HRQoL validation	Patients with cancer including hematological malignancies	Structural validity, internal consistency, construct validity
FACT-G	1993	D F Cella [50]	United States/English	Development and validation of general cancer HRQoL measure	Patients with cancer	PROM development, content validity, internal consistency, construct validity
MSAS	1994	R K Portenoy [51]	United States/English	Development of symptom assessment scale	Patients with cancer	PROM development, internal consistency, construct validity
ESAS	2000	V T Chang [52]	United States/English	Validation of symptom assessment system	Patients with cancer or palliative-care needs	Reliability, construct validity, responsiveness
MSAS-SF	2000	V T Chang [53]	United States/English	Short-form symptom assessment	Patients with cancer	Internal consistency, construct validity
EQ-5D-5 L	2011	M Herdman [54]	International/multilingual	Development of five-level EQ-5D	General and patient populations	PROM development, content validity, construct validity
EQ-5D-5 L	2012	Seon Ha Kim [55]	Korea/Korean	Validation of EQ-5D-5 L	Korean general/patient sample	Hypothesis testing for construct validity, including known-groups comparisons; feasibility
SF-36	1992	J E Brazier [56]	United Kingdom/English	Validation of generic health survey	General population/patient samples	Internal consistency; hypothesis testing for construct validity, including known-groups comparisons

Generic instruments were retained because they are commonly used as comparator or companion measures but were not interpreted as MM-specific PROMs

*PROM* patient-reported outcome measure, *MM* multiple myeloma, *HRQoL* health-related quality of life

### Characteristics of included PROMs

The included PROMs varied substantially in construct coverage, item number, recall period, scoring structure, and intended use. MM-specific modules, such as the EORTC QLQ-MY20, FACT-MM, MDASI-MM, MySIm-Q, and QLICP-MM, were designed to capture symptoms and impacts that are more directly relevant to MM and its treatment. MyPOS and HM-PRO covered broader palliative-care or hematological-malignancy concerns and included physical, emotional, informational, and practical domains. Generic instruments such as EQ-5D-5 L and SF-36 offer comparability across diseases but provide limited coverage of MM-specific symptoms and treatment-related burden. Instrument characteristics are presented in Table 2.

**Table 2:** Characteristics of included PROMs

PROM	Target construct	No. of items / domains	Recall period	Response/scoring	MM specificity	Typical use
EORTC QLQ-MY20 [32–38]	MM-specific symptoms and impacts	20 items; disease symptoms, side effects, future perspective, body image	Past week	4-point Likert; transformed to 0–100	MM-specific module	Use with EORTC QLQ-C30 in trials and observational HRQoL studies
MyPOS [39–41]	MM symptoms, concerns, palliative-care needs, practical and emotional impact	Multi-domain instrument; patient-centered concerns	Recent days/current concerns	Likert-type responses; domain/total scoring	MM-specific	Clinical/palliative-care assessment and research
FACT-MM [42]	General cancer HRQoL plus MM-specific concerns	FACT-G plus MM module	Past 7 days	5-point Likert; higher scores generally better	MM-specific module	Studies aligned with the FACIT framework
MDASI-MM [43, 44]	Symptom severity and interference	Core symptom and interference items plus MM-specific symptoms	Past 24 h	0–10 numeric rating	MM-specific symptom module	Symptom-burden assessment and treatment monitoring
MySIm-Q [45]	MM symptoms and impacts	Symptom and impact domains	Recent days/past week	Likert or numeric response options; domain scoring	MM-specific	Clinical trials focusing on symptom impact
QLICP-MM [18]	Quality of life in Chinese patients with MM	Core QLICP domains plus MM-specific module	Recent period	Likert-type responses; transformed scores	MM-specific module	Chinese-language HRQoL studies
HM-PRO [46–48]	Hematological malignancy symptoms, signs, and impacts	Part A impact items and Part B symptom/sign items	Recent period	Likert-type responses; part scores	Hematology-specific; includes MM	Hematological malignancy research and clinical assessment
EORTC QLQ-C30 [49]	Cancer-specific HRQoL	30 items; functioning, symptoms, global health	Past week	4-point and 7-point responses; 0–100 transformed scores	Cancer-specific, not MM-specific	Companion measure with QLQ-MY20
FACT-G [50]	General cancer HRQoL	27 items; physical, social/family, emotional, functional well-being	Past 7 days	5-point Likert; higher scores better	Cancer-specific, not MM-specific	Companion measure or framework for FACT-MM
MSAS [51]	Symptom prevalence, severity, frequency, and distress	32 symptoms and summary indices	Past week	Mixed symptom-specific response scales	Generic cancer symptom scale	Broad symptom assessment
ESAS [52]	Core symptom severity	Commonly 9–10 symptoms	Current/24 hours	0–10 numeric rating	Generic symptom scale	Clinical symptom screening
MSAS-SF [53]	Short-form symptom burden	Reduced item set and summary indices	Past week	Symptom-specific response options	Generic cancer symptom scale	Short symptom assessment
EQ-5D-5 L [54, 55]	Generic health status/utility	5 dimensions plus visual analogue scale	Today	5 levels per dimension; utility index and VAS	Generic	Health-economic evaluation and cross-disease comparison
SF-36 [56]	Generic physical and mental health	36 items; 8 domains and summary scores	Past 4 weeks or acute version	Domain scores 0–100	Generic	Cross-condition HRQoL comparison

Instrument details should be checked against the final author manuals before submission, especially recall period and scoring algorithms

### Methodological quality of included studies

The methodological quality of included studies differed across measurement properties. The strongest methodological evidence was generally observed for internal consistency and structural validity when studies reported adequate sample sizes and used appropriate factor-analytic methods. Content validity evidence was less consistently reported, particularly in studies that relied primarily on expert review or psychometric field testing without detailed patient involvement. Evidence for cross-cultural validity or measurement invariance was uncommon despite the availability of translated versions of several PROMs. Measurement error and criterion validity were rarely reported. The COSMIN risk-of-bias summary is shown in Table 3.

**Table 3:** COSMIN risk-of-bias summary by PROM and measurement property

PROM	PROM development	Content validity	Structural validity	Internal consistency	Reliability	Measurement error	Hypothesis testing for construct validity	Responsiveness	Main methodological concerns
EORTC QLQ-MY20 [32–38]	Adequate	Adequate/limited contemporary evidence	Adequate	Adequate	Adequate where reported	Not adequately reported	Adequate	Adequate in limited studies	Older development evidence; limited updated content validation and measurement-error evidence
MyPOS [39–41]	Adequate	Adequate	Adequate	Adequate	Adequate	Not adequately reported	Adequate	Adequate/ limited	Some samples small or context-specific; measurement error limited
FACT-MM [42]	Adequate	Adequate	Doubtful/ limited	Adequate	Not consistently reported	Not reported	Adequate/ limited	Not reported	Limited independent validation and structural evidence
MDASI-MM [43, 44]	Adequate	Adequate	Adequate	Adequate	Adequate	Not reported	Adequate	Limited/not reported	Primarily symptom-focused; limited responsiveness and measurement-error evidence
MySIm-Q [45]	Adequate	Adequate	Adequate	Adequate	Adequate	Not reported	Adequate	Limited/ not reported	Evidence concentrated in development/validation context
QLICP-MM [18]	Doubtful/adequate	Doubtful/limited	Adequate	Adequate	Adequate	Not reported	Adequate	Adequate/ limited	Mainly Chinese-language evidence; limited cross-cultural evidence
HM-PRO [46–48]	Adequate	Adequate	Adequate	Adequate	Adequate	Not reported	Adequate	Limited/not reported	Hematology-specific rather than MM-specific; MM subgroup evidence may be indirect
EORTC QLQ-C30 [49]	Adequate	Adequate for cancer, indirect for MM	Adequate	Adequate	Adequate	Limited	Adequate	Adequate in oncology	Content validity for MM-specific concerns is indirect
FACT-G [50]	Adequate	Adequate for cancer, indirect for MM	Adequate	Adequate	Adequate	Limited	Adequate	Limited	Not designed for MM-specific symptoms
MSAS [51]	Adequate	Adequate for cancer symptoms, indirect for MM	Adequate	Adequate	Limited	Not reported	Adequate	Limited	Symptom inventory, not HRQoL-specific
ESAS [52]	Adequate	Adequate for symptom screening, indirect for MM	Not applicable /limited	Not applicable/limited	Adequate/limited	Limited	Adequate	Adequate/ limited	Screening tool; limited multidimensional HRQoL coverage
MSAS-SF [53]	Adequate	Adequate for cancer symptoms, indirect for MM	Adequate	Adequate	Limited	Not reported	Adequate	Limited	Short form may reduce comprehensiveness
EQ-5D-5 L [54, 55]	Adequate	Adequate for generic health status, insufficient for MM-specific HRQoL alone	Not applicable	Not applicable	Adequate/limited	Limited	Adequate	Limited	Generic utility measure; content gap for MM-specific concerns
SF-36 [56]	Adequate	Adequate for generic health, insufficient for MM-specific HRQoL alone	Adequate	Adequate	Adequate/limited	Limited	Adequate	Limited	Generic measure; lacks MM-specific symptom and treatment-burden content

This table summarizes methodological quality at PROM level. In the final supplementary file, the same ratings can be expanded to study level. Known-groups comparisons were classified as one form of hypothesis testing for construct validity and were not treated as discriminant validity

### Content validity

Content validity was judged by considering relevance, comprehensiveness, and comprehensibility. PROMs developed specifically for MM or hematological malignancies generally had better conceptual relevance than generic HRQoL instruments. However, even among MM-specific PROMs, the depth of patient involvement and documentation of item-generation procedures varied. MyPOS, MySIm-Q, HM-PRO, and MDASI-MM provided relatively clearer evidence of patient input during development or content evaluation. The EORTC QLQ-MY20 showed broad clinical uptake and supportive validity evidence, but additional contemporary content-validation work may be needed to confirm comprehensiveness in the era of novel MM therapies. Generic instruments were considered useful as companion measures but were not judged sufficient on their own for comprehensive MM-specific HRQoL assessment.

### Structural validity and internal consistency

Structural validity was reported for several PROMs, most often through exploratory or confirmatory factor analysis. The EORTC QLQ-MY20, MyPOS, MDASI-MM, MySIm-Q, QLICP-MM, and HM-PRO generally showed interpretable domain structures, although the level of evidence varied by study design and sample size. Internal consistency was the most frequently reported measurement property. Most multi-item domains achieved acceptable reliability coefficients, but some short domains or domains with heterogeneous content had borderline estimates. Internal consistency ratings were interpreted together with structural validity, as recommended by COSMIN.

### Reliability, measurement error, and responsiveness

Test–retest reliability was available for only a subset of PROMs and was generally supportive when reported. However, the time interval between administrations and the evidence that patients were stable were not always sufficiently justified. Measurement-error evidence was largely unavailable, because few studies reported the standard error of measurement, smallest detectable change, limits of agreement, or minimally important change. Responsiveness was reported less frequently than internal consistency and construct validity. Where available, responsiveness evidence was mostly supportive but should be interpreted cautiously because longitudinal hypotheses and anchor-based interpretability thresholds were often incompletely specified.

### Hypothesis testing for construct validity

Hypothesis testing for construct validity was commonly evaluated through correlations with related instruments or comparisons between clinically defined groups. In COSMIN terminology, known-groups comparisons represent one form of hypothesis testing for construct validity. Discriminant validity, in contrast, refers to evidence that PROM scores are not strongly associated with measures of different constructs. Therefore, discriminant validity was not conflated with known-groups comparisons in this review. Evidence was generally sufficient when PROM scores differed in expected directions across clinical severity, treatment status, performance status, or symptom-burden groups. Nevertheless, many studies did not clearly predefine hypotheses regarding the expected direction and magnitude of associations, which downgraded the certainty of evidence.

### Overall evidence and recommendations

No PROM had high-certainty evidence for all key measurement properties in MM. The EORTC QLQ-MY20 had the broadest international evidence base and is the most practical MM-specific companion module when used with the EORTC QLQ-C30. MyPOS also showed relatively comprehensive evidence and may be particularly useful when palliative-care needs, symptom burden, and broader patient concerns are central outcomes. MDASI-MM and MySIm-Q are appropriate when symptom assessment is the primary objective, whereas FACT-MM and QLICP-MM may be considered when their scoring framework and language context match the study setting. Generic instruments such as EQ-5D-5 L and SF-36 are useful for health-economic evaluation or cross-condition comparison but should not be the sole PROM when MM-specific HRQoL is the main construct. The evidence synthesis and recommendations are summarized in Tables 4, 5, 6 and 7.

**Table 4:** Summary of measurement-property evidence and COSMIN recommendations

PROM	Content validity	Structural validity	Internal consistency	Reliability	Measurement error	Criterion validity	Construct validity / hypothesis testing	Responsiveness	Overall certainty	Recommendation
EORTC QLQ-MY20 [32–38]	+ / Low	+ / Low–Moderate	+ / Moderate	+ / Low	? / Very low	? / Very low	+ / Low–Moderate	+ / Low	Low–Moderate	A: strongest current MM-specific option, but update content validity and change metrics
MyPOS [39–41]	+ / Moderate	+ / Low	+ / Low–Moderate	+ / Low	? / Very low	? / Very low	+ / Low	+ / Low	Low–Moderate	A: suitable when broad patient concerns and palliative-care needs are central
FACT-MM [42]	+ / Low	? / Very low	+ / Low	? / Very low	? / Very low	? / Very low	+ / Low	? / Very low	Low	B: promising but needs independent structural, reliability, and responsiveness evidence
MDASI-MM [43, 44]	+ / Low–Moderate	+ / Low	+ / Low–Moderate	+ / Low	? / Very low	? / Very low	+ / Low	? / Very low	Low	B: appropriate for MM symptom burden, not stand-alone HRQoL
MySIm-Q [45]	+ / Moderate	+ / Low	+ / Low	+ / Low	? / Very low	? / Very low	+ / Low	? / Very low	Low	B: useful for symptom-impact endpoints; further independent validation and change-metric studies needed
QLICP-MM [18]	? / Low	+ / Low	+ / Low	+ / Low	? / Very low	? / Very low	+ / Low	+ / Low	Low	B: potentially useful in Chinese contexts; cross-cultural evidence needed
HM-PRO [46–48]	+ / Low–Moderate	+ / Low	+ / Low	+ / Low	? / Very low	? / Very low	+ / Low	? / Very low	Low	B: useful for hematological malignancies; MM-specific evidence indirect
EORTC QLQ-C30 [49]	+ / Moderate for cancer; indirect for MM	+ / Moderate	+ / Moderate	+ / Low	? / Very low	? / Very low	+ / Low	+ / Low	Moderate for cancer; low for MM specificity	B: companion measure; not sufficient alone for MM-specific HRQoL
FACT-G [50]	+ / Moderate for cancer; indirect for MM	+ / Moderate	+ / Moderate	+ / Low	? / Very low	? / Very low	+ / Low	? / Very low	Moderate for cancer; low for MM specificity	B: companion measure or framework for FACT-MM
MSAS [51]	+ / Moderate for symptoms; indirect for MM	+ / Low	+ / Low	? / Very low	? / Very low	? / Very low	+ / Low	? / Very low	Low	B: symptom companion measure
ESAS [52]	+ / Low for symptom screening; indirect for MM	N/A	N/A	+ / Low	? / Very low	? / Very low	+ / Low	+ / Low	Low	B: symptom screening only
MSAS-SF [53]	+ / Low for symptoms; indirect for MM	+ / Low	+ / Low	? / Very low	? / Very low	? / Very low	+ / Low	? / Very low	Low	B: brief symptom companion measure
EQ-5D-5 L [54, 55]	+ / Moderate for generic health; indirect for MM	N/A	N/A	+ / Low	? / Very low	? / Very low	+ / Low	? / Very low	Low for MM-specific HRQoL	B: health-economic companion measure only
SF-36 [56]	+ / Moderate for generic health; indirect for MM	+ / Moderate	+ / Moderate	+ / Low	? / Very low	? / Very low	+ / Low	? / Very low	Low for MM-specific HRQoL	B: generic comparator, not MM-specific

+ = sufficient; − = insufficient; ? = indeterminate; N/A = not applicable. Certainty ratings are expressed as result/certainty. Recommendation A = sufficient content validity + at least low‑quality evidence for sufficient internal consistency; B = promising but requires further validation

**Table 5:** Practical interpretation and feasibility of included PROMs

PROM	Strengths	Limitations	Best suited for	Use recommendation
EORTC QLQ-MY20	Most widely evaluated MM-specific module; compatible with EORTC QLQ-C30; international use	Older development evidence; limited measurement error and contemporary content validation	MM-specific HRQoL in trials and observational studies	Preferred MM-specific HRQoL module when EORTC framework is used
MyPOS	Patient-centered; broad coverage of symptoms, emotional concerns, practical issues, and palliative-care needs	Less widely used than QLQ-MY20; measurement error limited	Clinical and palliative-care MM research	Strong option when comprehensive patient concerns are central
FACT-MM	Built on FACIT/FACT-G framework; MM-specific module	Less evidence for structural validity and responsiveness	Studies already using FACT instruments	Use when FACIT compatibility is needed; collect additional validation data
MDASI-MM	Brief symptom-severity and interference measure; intuitive numeric ratings	Symptom-focused and not comprehensive HRQoL	Symptom burden, treatment toxicity, clinical monitoring	Use as symptom endpoint or companion measure
MySIm-Q	Developed for MM symptoms and impacts; patient-centered symptom-impact focus	Independent validation and interpretability/MIC evidence remain limited	Symptom-impact endpoints in trials	Promising; further validation recommended
QLICP-MM	Designed for Chinese MM population; disease-specific module	Limited international evidence and cross-cultural validation	Chinese-language HRQoL studies	Use in Chinese contexts; expand validation
HM-PRO	Hematology-specific broad instrument; includes symptoms and impacts	Not MM-specific; MM subgroup evidence may be indirect	Hematological malignancy studies including MM	Use when cross-hematology comparison is desired
EORTC QLQ-C30	Established cancer HRQoL core instrument; compatible with QLQ-MY20	Not MM-specific	Core cancer HRQoL companion measure	Use with QLQ-MY20 rather than alone
FACT-G	Established cancer HRQoL core instrument; compatible with FACT-MM	Not MM-specific	FACIT/FACT framework studies	Use with FACT-MM rather than alone
MSAS/MSAS-SF	Captures multidimensional symptom burden	Not MM-specific; not HRQoL comprehensive	Broad symptom assessment	Use as symptom companion measure
ESAS	Very brief symptom screening; feasible in clinical settings	Limited domains; screening rather than comprehensive measurement	Routine symptom screening	Use for screening, not primary HRQoL endpoint
EQ-5D-5 L	Utility scores for cost-effectiveness; low burden	Major content gaps for MM-specific symptoms	Economic evaluation and cross-disease comparison	Use only as companion utility measure
SF-36	Broad generic health profile; cross-condition comparison	Limited MM-specific sensitivity	General health comparison	Use as generic comparator, not stand-alone MM HRQoL measure

This table supports clinical and research selection but does not replace the COSMIN evidence ratings in Table 4

**Table 6:** Final recommended PROM selection framework for MM research and clinical assessment

Rank/role	Recommended PROM(s)	Rationale	Important caveat
Primary MM-specific HRQoL assessment	EORTC QLQ-MY20 + EORTC QLQ-C30	Broadest MM-specific evidence base; international use; compatible oncology core questionnaire	Update content validity and change metrics are still needed
Broad patient concerns and palliative-care needs	MyPOS	Patient-centered coverage of symptoms, emotional, practical, informational, and care-related concerns	Less evidence than QLQ-MY20 in some psychometric domains
Symptom-burden endpoint	MDASI-MM; MySIm-Q	Designed to capture MM symptom severity, interference, and symptom impacts	Not sufficient for comprehensive HRQoL alone
Chinese-language MM-specific HRQoL	QLICP-MM; Chinese QLQ-MY20; Chinese MDASI-MM	Available Chinese-language options with supportive evidence	Cross-cultural comparability and measurement invariance require further testing
Hematological malignancy comparison	HM-PRO	Useful when MM is studied alongside other hematologic malignancies	MM-specific content may be less complete than disease-specific instruments
Health-economic or cross-disease comparison	EQ-5D-5 L; SF-36	Provide utility or generic health estimates	Should be companion measures only when MM-specific HRQoL is the main construct

Final selection should also consider respondent burden, licensing, language availability, scoring requirements, target construct, and whether the study requires disease-specific or generic comparability

**Table 7:** Final recommendation for each PROM

PROM	Recommendation class	Basis for recommendation	Suggested use	Key caveats/evidence gaps
QLQ-MY20	A	Sufficient content validity and moderate-certainty evidence for internal consistency; additional supportive evidence was available for reliability, hypothesis testing for construct validity, and responsiveness.	Preferred disease-specific module for MM HRQoL assessment, especially in trials or studies using the EORTC system	Must be administered with QLQ-C30 core questionnaire; evidence gaps remain for measurement error and formal measurement invariance
MyPOS	A	Moderate-certainty evidence supported content validity, and low-to-moderate evidence supported internal consistency; reliability, hypothesis testing for construct validity, and responsiveness were also supported by low-certainty evidence.	Suitable for routine clinical monitoring and supportive-care-oriented assessment in MM	Healthcare Support subscale and floor effects require attention; measurement error evidence lacking
MDASI-MM	B	Content validity, structural validity, internal consistency, reliability, and hypothesis testing for construct validity were supported, but evidence for measurement error, criterion validity, and responsiveness remained limited or indeterminate.	Appropriate when the primary purpose is symptom burden and symptom interference assessment	Not a full HRQoL instrument; measurement error and cross-cultural/MI evidence remain limited
HM-PRO	B	Content validity, structural validity, internal consistency, reliability, and hypothesis testing for construct validity were supported, but the evidence was partly indirect because HM-PRO is hematology-specific rather than MM-specific.	Can be considered when a generic hematologic malignancy PROM is needed	Important indirectness (evidence from hematological malignancies, not MM-specific); MM‑specific subgroup evidence should be strengthened.
FACT-G	B	A core cancer PROM with supportive evidence for structural validity, internal consistency, reliability, and hypothesis testing for construct validity, although content validity evidence was indirect for MM-specific HRQoL.	Useful for general cancer HRQoL comparison, especially alongside disease-specific modules	Indirectness for MM (evidence from general cancer populations); does not cover myeloma‑specific issues alone.
FACT-MM	B	Low-certainty evidence supported content validity, internal consistency, and hypothesis testing for construct validity, whereas evidence for structural validity, reliability, measurement error, criterion validity, and responsiveness remained limited or indeterminate.	Promising MM-specific module; may be used when FACT system is required	Needs independent validation in larger MM samples, particularly for structural validity, reliability, measurement error, responsiveness, and interpretability.
MySIm-Q	B	Content validity, structural validity, internal consistency, reliability, and hypothesis testing for construct validity were supported, but evidence for measurement error, criterion validity, responsiveness, and interpretability/MIC remained limited or indeterminate.	Promising instrument for active MM symptom/impact measurement in trials	Further studies are needed to examine measurement error, responsiveness, criterion validity where appropriate, and interpretability/MIC.
QLICP-MM	B	Content validity was partly indeterminate, whereas structural validity, internal consistency, reliability, hypothesis testing for construct validity, and responsiveness showed supportive but low-certainty evidence.	Potentially useful in Chinese MM populations	Cannot receive A-level until content-validity evidence is clarified and broader cross-cultural and measurement-error evidence becomes available.
QLQ-C30	B	A core cancer PROM with supportive evidence for structural validity, internal consistency, reliability, hypothesis testing for construct validity, and responsiveness, although content validity evidence was indirect for MM-specific HRQoL.	Should be used as the EORTC core questionnaire with QLQ-MY20	B reflects review evidence, not global legacy evidence
MSAS	B	Content validity for cancer symptoms was indirect for MM; structural validity, internal consistency, and hypothesis testing for construct validity showed low-certainty support, whereas reliability, measurement error, and responsiveness remained limited or indeterminate.	Use for multidimensional symptom assessment rather than HRQoL alone	Not MM-specific; content validity and measurement error gaps remain
ESAS	B	Brief symptom-screening measure with supportive reliability, hypothesis-testing, and responsiveness evidence in cancer or palliative-care populations; internal consistency and structural validity were not treated as primary criteria for this item-level symptom tool.	Useful for rapid symptom screening	Composite symptom tool; internal consistency/structural validity should be interpreted cautiously
MSAS-SF	B	A brief symptom instrument with low-certainty evidence supporting structural validity, internal consistency, and hypothesis testing for construct validity; reliability, measurement error, and responsiveness remained limited or indeterminate.	Use where a shorter symptom burden tool is needed	Not MM-specific; content validity and measurement error gaps remain
EQ-5D-5 L	B	Content validity and reliability were supported for generic/preference-based use, and hypothesis testing for construct validity showed low-certainty support; structural validity and internal consistency were not treated as applicable.	Useful when health utility or economic evaluation is needed	Preference-based formative/composite instrument; use COSMIN reflective criteria cautiously
SF-36	B	Generic legacy measure with supportive evidence for structural validity, internal consistency, reliability, and hypothesis testing for construct validity, but content validity evidence was indirect for MM-specific HRQoL.	Use for broad generic health status comparisons	B reflects MM-focused evidence base; global legacy evidence may be underestimated

A = PROMs that can be recommended for use; B = PROMs with potential use but requiring further validation

## Discussion

### Main findings

This COSMIN-based systematic review identified a heterogeneous body of evidence for PROMs used to assess HRQoL, symptoms, and disease burden in MM. The findings suggest that several MM-specific or MM-relevant instruments are available, but the psychometric evidence base is uneven across measurement properties. Most validation studies focused on internal consistency, structural validity, and construct validity, whereas content validity, measurement error, cross-cultural validity, interpretability, and responsiveness were less frequently and less rigorously addressed.

A central finding is that instrument specificity matters. PROMs developed for MM or hematological malignancies were more likely to cover symptoms and impacts that are highly salient to patients with MM, such as bone pain, fatigue, treatment-related adverse effects, uncertainty about the future, and disease-specific functional limitations. However, disease specificity alone should not be interpreted as sufficient psychometric evidence. According to COSMIN, content validity requires explicit evidence that items are relevant, comprehensive, and comprehensible to the target population. Therefore, instruments with strong clinical uptake, such as the EORTC QLQ-MY20, still benefit from updated content-validation work, particularly because MM treatment pathways and survivorship experiences have changed substantially over time.

The EORTC QLQ-MY20 emerged as the most extensively studied MM-specific module. Its major strengths are broad international use, compatibility with the EORTC QLQ-C30, and supportive evidence for several measurement properties. This makes it a defensible option for MM clinical trials and observational studies, especially when comparison with oncology HRQoL data is needed. MyPOS also demonstrated a relatively broad evidence base and may be particularly useful in settings where symptom burden, palliative-care concerns, emotional distress, information needs, and practical problems are all important. MDASI-MM and MySIm-Q provide more symptom-focused alternatives, while FACT-MM and QLICP-MM may be useful depending on the study context, language, and scoring infrastructure.

A second important finding is that several key COSMIN domains remain underdeveloped. Measurement-error evidence was rarely reported, which limits interpretation of individual-level change and the ability to distinguish true change from random variation. Responsiveness evidence was also limited and often lacked prespecified hypotheses or anchor-based thresholds. Cross-cultural validity and measurement invariance were seldom tested, even when translated versions of instruments were used. These gaps are especially important in MM research because international trials, multinational registries, and cross-cultural survivorship studies increasingly rely on harmonized PROM data.

The results also clarify the role of generic instruments. EQ-5D-5 L and SF-36 are useful for economic evaluation, burden-of-disease comparisons, and cross-condition benchmarking. Nevertheless, their content validity for MM-specific HRQoL is limited because they do not fully capture symptoms, treatment toxicities, and psychosocial concerns that are central to MM patients. Therefore, they should be used as companion measures rather than substitutes for MM-specific PROMs when the primary outcome is MM-related HRQoL.

#### Psychometric properties

Although various instruments are available for precise measurement of QoL in MM patients, most PROMs included in this review lack comprehensive evidence across a range of psychometric properties. Content validity is the most crucial measurement property for PROMs, reflecting the degree to which an instrument’s content adequately represents the construct it intends to measure [31]. In this systematic review, most studies focused on relevance and comprehensibility, interviewing experts and/or patients, but few conducted formal expert consultation, and comprehensiveness was often neglected. The assessment of content validity can be based on either qualitative methods (e.g., interviews, cognitive interviews) or quantitative methods (e.g., importance ratings), each of which has its own value. Among the studies included in this review, some employed mixed methods while others used only a single method; this does not constitute a methodological flaw. Therefore, future studies must employ more rigorous and transparent methods (e.g., conducting adequate cognitive interviews with detailed reporting) to re-evaluate the content validity of existing tools.

Structural validity assesses whether a tool measures the intended dimensions. Results showed variable methodological quality. Confirmatory Factor Analysis (CFA), a superior method for validating hypothesized structures [57], was used in only 4 studies [35, 40, 44, 48], while more studies employed Exploratory Factor Analysis (EFA) or Principal Component Analysis (PCA). Multiple EFA/PCA studies were rated “insufficient” (-) due to a high number of cross-loadings (> 10% of items loading ≥ 0.30 on multiple factors), suggesting unclear internal structure or multidimensionality, potentially affecting interpretative accuracy. Therefore, instruments with poor results from EFA/PCA (e.g., FACT-MM, MySIm-Q) require further validation using CFA in independent samples. Advanced methods like Rasch analysis or confirmatory bifactor models should be used to examine unidimensionality, providing a more solid basis for assessing internal consistency [58, 59]. Internal consistency is a key indicator of the homogeneity among items within a tool [27]. In this review, most studies assessed this property, with generally good methodological quality and sufficient measurement properties, indicating these instruments have acceptable internal reliability at the total or subscale level. Test-retest reliability assesses score stability over time. Ten studies [18, 35, 36, 38, 40, 50, 52, 53, 55, 56] evaluated this, but half lacked a theoretical basis for retest interval selection (too short or unspecified), and others failed to ensure stable patient health status or consistent conditions between tests, potentially leading to over- or underestimation of reliability [60]. COSMIN guidelines suggest a suitable retest interval for relatively stable constructs like QoL is typically 2–4 weeks [61]. Future reliability studies should design retest procedures rigorously, specify the interval clearly, and assess and report the conditions and patient status at retest.

Hypothesis testing is a core method for assessing the construct validity of PROMs [25]. In this review, 16 studies [18, 32–34, 36, 37, 39, 43, 44, 47, 50–53, 55, 56] provided supportive evidence through hypothesis testing, including correlations with related constructs and comparisons between clinically distinct groups. Most of these studies reported that PROM scores differed in expected directions across groups defined by disease status, treatment phase, performance status, or symptom burden, providing supportive known-groups evidence. In COSMIN terminology, known-groups comparisons are best interpreted as one form of hypothesis testing for construct validity rather than as discriminant validity. Discriminant validity, in contrast, refers to evidence that PROM scores are not strongly associated with measures of different constructs. However, three studies [18, 44, 47] did not report subgroup comparisons. This reporting gap limits the interpretation of whether these instruments can distinguish clinically meaningful patient groups in real-world MM settings [27]. Insufficient known-groups evidence may also reduce the practical value of a PROM for guiding individualized treatment decisions or identifying high-risk patient groups [62]. Future studies should predefine hypothesis-testing criteria, including expected directions and magnitudes of correlations or group differences, and should select clinically meaningful contrast groups such as newly diagnosed versus relapsed/refractory patients or patients with different treatment responses [63].

Responsiveness is crucial for monitoring patient change over time and for evaluating treatment effects in clinical trials and longitudinal studies [64]. Responsiveness evidence was reported less frequently than internal consistency and construct validity. Where available, responsiveness evidence was mostly supportive, but the certainty remained low because longitudinal hypotheses, stable anchors, and anchor-based interpretability thresholds were often incompletely specified. With the rapid evolution of MM treatments, more longitudinal studies are needed. Future studies should employ anchor-based methods, such as the Patient Global Impression of Change, and distribution-based methods where appropriate; predefine hypotheses regarding expected score changes; and estimate or prespecify minimal important change values to evaluate whether PROMs can detect true change over time. Such evidence would help determine PROM sensitivity to treatment response, disease relapse, or symptom worsening [65].

### Cross-cultural validity, measurement-error evidence, and criterion validity: underreported dimensions

This review found no included studies that formally assessed cross-cultural validity or measurement invariance. Most cross-cultural studies were limited to translation and linguistic adaptation and did not statistically test measurement equivalence, such as factor structure, item functioning, or differential item functioning [66, 67]. Similarly, no included study provided a formal COSMIN-based evaluation of measurement error using indices such as the standard error of measurement, smallest detectable change, limits of agreement, or comparison with a minimal important change. This lack of measurement-error evidence limits interpretation of individual-level score changes, because it remains difficult to determine whether an observed change reflects true change rather than random measurement variation [68]. Regarding criterion validity, we acknowledge that for most HRQoL PRO measures, no true gold standard exists [69]. Objective clinical outcomes, such as progression-free survival or laboratory biomarkers, should not be automatically treated as criteria for patient-reported HRQoL. Instead, they may serve as external clinical anchors or known-group variables only when a clear theoretical rationale links them to the PRO construct [70]. In the absence of both a gold standard and theory-grounded anchors, traditional criterion validity could not be meaningfully assessed in the reviewed studies. Future validation efforts should strengthen hypothesis-testing evidence, including known-groups comparisons, convergent validity, and discriminant validity when appropriate measures of dissimilar constructs are available, and should examine associations with relevant clinical endpoints only as supporting evidence when theoretically justified [27].

#### Implications for clinical practice

Multiple myeloma and its treatment impose a persistent and multidimensional burden on patients’ physical function, psychological state, and overall quality of life. Therefore, systematic and precise assessment of patient-reported outcomes is crucial for implementing individualized support, optimizing symptom management, and enhancing overall care quality. Based on the synthesized evidence from this systematic review following COSMIN guidelines, we suggest clinicians and researchers prioritize instruments with an A-level recommendation based on specific assessment purposes. For MM-specific HRQoL assessment, the EORTC QLQ-MY20 (used with the QLQ-C30) and MyPOS are the current A-level options. For symptom-focused purposes, B-level instruments such as MDASI-MM may be considered, but they require further validation. However, no single instrument is superior in all measurement properties. In clinical practice, factors such as completion time, patient burden, and the availability of validated localized language versions must also be weighed when selecting an instrument.

#### Limitations and future directions

This study followed PRISMA-COSMIN guidelines [27], involved two independent reviewers, and used updated COSMIN standards for evidence grading and synthesis, yielding reliable conclusions. However, limitations include the near-universal absence of evidence for cross-cultural validity. This implies that although the QLQ-MY20 received an A-level recommendation in this review, its version in China or non-English-speaking countries may not have undergone rigorous measurement invariance testing (e.g., DIF analysis); thus, cross-country comparative data should be interpreted cautiously. Limiting the search to English and Chinese literature may have missed important validation studies from Europe (e.g., in German, French) on EORTC instruments. Incomplete reporting in some original studies affected methodological quality ratings. Grey literature (e.g., unpublished pharmaceutical industry reports) was not searched, potentially introducing publication bias. Downgrading for “indirectness” in evidence grading reflects that some instruments were not specifically developed for MM. While this aligns with COSMIN standards, it also suggests the application value of generic instruments in specific populations needs to be considered alongside clinical judgment. Furthermore, this review only included psychometric validation studies conducted in patients with multiple myeloma. Therefore, a substantial body of evidence on measurement properties (particularly cross-cultural equivalence and longitudinal measurement invariance) for generic or cross-cancer instruments such as the SF-36, EORTC QLQ-C30, and FACT-G, which was accumulated in other populations (e.g., other cancers, chronic diseases), was not included. This may lead to an underestimation of the overall performance of these instruments.

Future MM studies should make reference to this existing evidence base whenever possible. Based on the above discussion, future research should: Prioritize high-quality cross-cultural validation: Systematic assessment of measurement invariance is essential when introducing an instrument into a new cultural context [71]. Adopt longitudinal designs: To comprehensively evaluate responsiveness, measurement error, and long-term reliability. Enhance methodological rigor: When using PROMs developed under a reflective measurement model, it is recommended to follow the COSMIN guidelines to improve study quality and comparability; for formative or composite measurement tools, applicable items should be selected with caution, and other methodological frameworks (such as specific criteria for health utility instruments) should be supplemented. Explore electronic migration: With the growing use of telemedicine in hematology management, validating the equivalence of migrating instruments from paper to electronic formats (apps, web-based) is a new focus. This review found very few studies specifically assessing the usability of electronic versions in the MM population. Future research should focus on the user experience (UI/UX) and feasibility of data collection for ePROMs, and assess measurement equivalence between paper-based PROMs and ePROMs to leverage the advantages of digital health [72]. Focus on clinical utility: In validation studies, combine anchor-based methods to determine thresholds for clinically meaningful change, facilitating the translation of research findings into clinical decision-making [73].

## Conclusion

The available evidence indicates that PROM selection in MM should be guided by the intended construct, study purpose, target population, and COSMIN-based evidence profile. The EORTC QLQ-MY20 currently has the broadest international evidence base for MM-specific HRQoL assessment, particularly when combined with the EORTC QLQ-C30. MyPOS is also well suited for capturing broad patient concerns and palliative-care needs. Symptom-focused measures such as MDASI-MM and MySIm-Q may be appropriate when symptom burden is the primary endpoint. Generic instruments remain valuable as companion measures but are insufficient as stand-alone instruments for MM-specific HRQoL. Further high-quality studies are needed to strengthen evidence for content validity, measurement error, cross-cultural validity, responsiveness, and interpretability.

## Supplementary Information

Supplementary Material 1.

## Data Availability

Data sharing is not applicable to this article as no datasets were generated or analysed during the current study.

## References

[1] Cowan AJ, Green DJ, Kwok M, Lee S, Coffey DG, Holmberg LA, Tuazon S, Gopal AK, Libby EN. Diagnosis and Management of Multiple Myeloma. Rev JAMA. 2022;327(5):464–77. 10.1001/jama.2022.0003. 10.1001/jama.2022.0003 35103762

[2] Moreau P, Touzeau C. (2015). Multiple myeloma: from front-line to relapsed therapies. American Society of Clinical Oncology educational book. American Society of Clinical Oncology. Annual Meeting, e504–e511. 10.14694/EdBook_AM.2015.35.e504 10.14694/EdBook_AM.2015.35.e504 25993216

[3] Kumar SK, Callander NS, Adekola K, Anderson LD, Baljevic M, Baz R, Campagnaro E, Costello C, D’Angelo C, Derman B, Devarakonda S, Elsedawy N, Godara A, Godby K, Hillengass J, Holmberg L, Htut M, Huff CA, Hultcrantz M, Kang Y, Kumar R. NCCN Guidelines^®^ Insights: Multiple Myeloma, Version 1.2025. J Natl Compr Cancer Network: JNCCN. 2025;23(5):132–40. 10.6004/jnccn.2025.0023. 40340857 10.6004/jnccn.2025.0023

[4] O’Donnell EK, Shapiro YN, Yee AJ, Nadeem O, Laubach JP, Branagan AR, Anderson KC, Mo CC, Munshi NC, Ghobrial IM, Sperling AS, Agyemang EA, Burke JN, Harrington CC, Hu BY, Richardson PG, Raje NS, El-Jawahri A. Quality of life, psychological distress, and prognostic perceptions in caregivers of patients with multiple myeloma. Blood Adv. 2022;6(17):4967–74. 10.1182/bloodadvances.2022007127. 35848842 10.1182/bloodadvances.2022007127 PMC9631626

[5] Kamal M, Wang XS, Shi Q, Zyczynski TM, Davis C, Williams LA, Lin HK, Garcia-Gonzalez A, Cleeland CS, Orlowski R. Symptom burden and its functional impact in patients with symptomatic relapsed or refractory multiple myeloma. Supportive care cancer: official J Multinational Association Supportive Care Cancer. 2021;29(1):467–75. 10.1007/s00520-020-05493-y. 10.1007/s00520-020-05493-y 32390093

[6] U.S. Department of Health and Human Services FDA Center for Drug Evaluation and Research, U.S. Department of Health and Human Services FDA Center for Biologics Evaluation and Research, & U.S. Department of Health and Human Services FDA Center for Devices and Radiological Health. Guidance for industry: patient-reported outcome measures: use in medical product development to support labeling claims: draft guidance. Health Qual Life Outcomes. 2006;4:79. 10.1186/1477-7525-4-79. 17034633 10.1186/1477-7525-4-79 PMC1629006

[7] Basch E, Deal AM, Kris MG, Scher HI, Hudis CA, Sabbatini P, Rogak L, Bennett AV, Dueck AC, Atkinson TM, Chou JF, Dulko D, Sit L, Barz A, Novotny P, Fruscione M, Sloan JA, Schrag D. Symptom Monitoring With Patient-Reported Outcomes During Routine Cancer Treatment: A Randomized Controlled Trial. J Clin oncology: official J Am Soc Clin Oncol. 2016;34(6):557–65. 10.1200/JCO.2015.63.0830. 10.1200/JCO.2015.63.0830 PMC4872028 26644527

[8] Efficace F, Gaidano G, Lo-Coco F. Patient-reported outcomes in hematology: is it time to focus more on them in clinical trials and hematology practice? Blood. 2017;130(7):859–66. 10.1182/blood-2017-03-737403. 28694324 10.1182/blood-2017-03-737403

[9] Dimopoulos MA, Moreau P, Terpos E, Mateos MV, Zweegman S, Cook G, Delforge M, Hájek R, Schjesvold F, Cavo M, Goldschmidt H, Facon T, Einsele H, Boccadoro M, San-Miguel J, Sonneveld P, Mey U. EHA Guidelines Committee. Electronic address: guidelines@ehaweb.org, & ESMO Guidelines Committee. Electronic address: clinicalguidelines@esmo.org (2021). Multiple myeloma: EHA-ESMO Clinical Practice Guidelines for diagnosis, treatment and follow-up†. Annals oncology: official J Eur Soc Med Oncol, 32(3), 309–22. 10.1016/j.annonc.2020.11.014 10.1016/j.annonc.2020.11.014 33549387

[10] Sonneveld P, Avet-Loiseau H, Lonial S, Usmani S, Siegel D, Anderson KC, Chng WJ, Moreau P, Attal M, Kyle RA, Caers J, Hillengass J, San Miguel J, van de Donk NW, Einsele H, Bladé J, Durie BG, Goldschmidt H, Mateos MV, Palumbo A, Orlowski R. Treatment of multiple myeloma with high-risk cytogenetics: a consensus of the International Myeloma Working Group. Blood. 2016;127(24):2955–62. 10.1182/blood-2016-01-631200. 27002115 10.1182/blood-2016-01-631200 PMC4920674

[11] Ullrich CK, Baker KK, Carpenter PA, Flowers ME, Gooley T, Stevens S, Krakow EF, Oshima MU, Salit RB, Vo P, Connelly-Smith L, Lee SJ, Wood WA. Fatigue in Hematopoietic Cell Transplantation Survivors: Correlates, Care Team Communication, and Patient-Identified Mitigation Strategies. Transplantation Cell therapy. 2023;29(3):e2001–8. 10.1016/j.jtct.2022.11.030. 10.1016/j.jtct.2022.11.030 36494015

[12] Boquoi A, Giagounidis A, Goldschmidt H, Heinsch M, Rummel MJ, Kröger N, Mai EK, Strapatsas J, Haas R, Kobbe G. Health-Related Quality of Life in Multiple Myeloma Patients Treated with High- or Low-Dose Lenalidomide Maintenance Therapy after Autologous Stem Cell Transplantation-Results from the LenaMain Trial (NCT00891384). Cancers. 2023;15(21):5157. 10.3390/cancers15215157. 37958331 10.3390/cancers15215157 PMC10650513

[13] Dixit J, Gupta N, Kataki A, Roy P, Mehra N, Kumar L, Singh A, Malhotra P, Gupta D, Goyal A, Rajsekar K, Krishnamurthy MN, Gupta S, Prinja S. Health-related quality of life and its determinants among cancer patients: evidence from 12,148 patients of Indian database. Health Qual Life Outcomes. 2024;22(1):26. 10.1186/s12955-024-02227-0. 38481231 10.1186/s12955-024-02227-0 PMC10938809

[14] Pedersen M, Larsen MT, Kornblit BT, Dahl EO, Lomborg K, Tolver A, Jarden M. Effects of nurse-led symptom management in chronic myeloid malignancies: a randomized trial. Supportive care cancer: official J Multinational Association Supportive Care Cancer. 2025;33(3):196. 10.1007/s00520-025-09230-1. 10.1007/s00520-025-09230-1 PMC11829834 39954038

[15] Forde K, Cocks K, Wells JR, McMillan I, Kyriakou C, EORTC Quality of Life Group. Use of the European Organisation for Research and Treatment of Cancer multiple myeloma module (EORTC QLQ-MY20): a review of the literature 25 years after development. Blood cancer J. 2023;13(1):79. 10.1038/s41408-023-00815-9. 37193682 10.1038/s41408-023-00815-9 PMC10188493

[16] Samala RV, Nurse DP, Chen X, Wei W, Crook JJ, Fada SD, Valent J. Effects of early palliative care integration on patients with newly diagnosed multiple myeloma. Supportive care cancer: official J Multinational Association Supportive Care Cancer. 2024;32(7):468. 10.1007/s00520-024-08665-2. 10.1007/s00520-024-08665-2 38937310

[17] Mina R, Mylin AK, Yokoyama H, Magen H, Alsdorf W, Minnema MC, Shune L, Isufi I, Harrison SJ, Shah UA, Schecter JM, Vogel M, Lendvai N, Gries KS, Katz EG, Slaughter A, Lonardi C, Gilbert J, Li Q, Deraedt W, Weisel K. Patient-reported outcomes following ciltacabtagene autoleucel or standard of care in patients with lenalidomide-refractory multiple myeloma (CARTITUDE-4): results from a randomised, open-label, phase 3 trial. Lancet Haematol. 2025;12(1):e45–56. 10.1016/S2352-3026(24)00320-X. 39756844 10.1016/S2352-3026(24)00320-X PMC12321245

[18] Li Weiqiang. (2022). Development of Quality of life measurement scale QLICP-MM (V2.0) for patients with multiple myeloma and Establishment of its Minimal Clinically Important Differences, Doctoral dissertation, Guangdong Medical University.

[19] Beer H, Chung H, J Harrison S, Quach H, Taylor-Marshall R, Jones L, Krishnasamy M. Listening to What Matters Most: Consumer Endorsed Patient Reported Outcome Measures (PROMs) for Use in Multiple Myeloma Clinical Trials: A Descriptive Exploratory Study. Clin lymphoma myeloma Leuk. 2023;23(7):505–14. 10.1016/j.clml.2023.03.008. 37087351 10.1016/j.clml.2023.03.008

[20] Weisel K, Dimopoulos MA, San-Miguel J, Paner A, Engelhardt M, Taylor F, Lord-Bessen J, Yip C, Greenwood M, Tang J, Cavo M. Impact of Elotuzumab Plus Pomalidomide/Dexamethasone on Health-related Quality of Life for Patients With Relapsed/Refractory Multiple Myeloma: Final Data From the Phase 2 ELOQUENT-3 Trial. HemaSphere. 2023;7(3):e843. 10.1097/HS9.0000000000000843. 36860268 10.1097/HS9.0000000000000843 PMC9970270

[21] Tremblay G, Daniele P, Breeze J, Li L, Shah J, Shacham S, Kauffman M, Engelhardt M, Chari A, Nooka A, Vogl D, Gavriatopoulou M, Dimopoulos MA, Richardson P, Biran N, Siegel D, Vlummens P, Doyen C, Facon T, Mohty M, Sundar J. Quality of life analyses in patients with multiple myeloma: results from the Selinexor (KPT-330) Treatment of Refractory Myeloma (STORM) phase 2b study. BMC Cancer. 2021;21(1):993. 10.1186/s12885-021-08453-9. 34488662 10.1186/s12885-021-08453-9 PMC8419947

[22] Du H, Jiao Q, Liu C, Wu X, Guo J, Zheng H, Zhao L. Dynamic Changes in Symptom Clusters and Symptom Networks in Patients With Multiple Myeloma: A Cross-lagged Network Analysis. Cancer Nurs. 2025. 10.1097/NCC.0000000000001537. Advance online publication. 40952868 10.1097/NCC.0000000000001537

[23] Chen F, Leng Y, Ni J, Niu T, Zhang L, Li J, Zheng Y. Symptom clusters and quality of life in ambulatory patients with multiple myeloma. Supportive care cancer: official J Multinational Association Supportive Care Cancer. 2022;30(6):4961–70. 10.1007/s00520-022-06896-9. 10.1007/s00520-022-06896-9 35182229

[24] Ebert RPC, Magnus MM, Toro P, Manoel FG, Costa FF, Saad O, S. T., de Melo Campos P. Hematologic Malignancies Patients Face High Symptom Burden and Are Lately Referred to Palliative Consultation: Analysis of a Single Center Experience. Am J Hosp palliat Care. 2023;40(7):761–4. 10.1177/10499091221132285. 36205034 10.1177/10499091221132285

[25] Prinsen CAC, Mokkink LB, Bouter LM, Alonso J, Patrick DL, de Vet HCW, Terwee CB. Qual life research: Int J Qual life aspects Treat care rehabilitation. 2018;27(5):1147–57. 10.1007/s11136-018-1798-3. COSMIN guideline for systematic reviews of patient-reported outcome measures. 10.1007/s11136-018-1798-3 PMC5891568 29435801

[26] Mokkink LB, de Vet HCW, Prinsen CAC, Patrick DL, Alonso J, Bouter LM, Terwee CB. Qual life research: Int J Qual life aspects Treat care rehabilitation. 2018;27(5):1171–9. 10.1007/s11136-017-1765-4. COSMIN Risk of Bias checklist for systematic reviews of Patient-Reported Outcome Measures. 10.1007/s11136-017-1765-4 PMC5891552 29260445

[27] Mokkink LB, Elsman EBM, Terwee CB. Int J Qual life aspects Treat care rehabilitation. 2024;33(11):2929–39. 10.1007/s11136-024-03761-6. COSMIN guideline for systematic reviews of patient-reported outcome measures version 2.0. Quality of life research:. 10.1007/s11136-024-03761-6 PMC11541334 39198348

[28] Elsman EBM, Mokkink LB, Terwee CB, Beaton D, Gagnier JJ, Tricco AC, Baba A, Butcher NJ, Smith M, Hofstetter C, Aiyegbusi OL, Berardi A, Farmer J, Haywood KL, Krause KR, Markham S, Mayo-Wilson E, Mehdipour A, Ricketts J, Szatmari P, Offringa M. Guideline for reporting systematic reviews of outcome measurement instruments (OMIs): PRISMA-COSMIN for OMIs 2024. J patient-reported outcomes. 2024;8(1):64. 10.1186/s41687-024-00727-7. 10.1186/s41687-024-00727-7 PMC11231111 38977535

[29] Terwee CB, Jansma EP, Riphagen II, de Vet HC. Development of a methodological PubMed search filter for finding studies on measurement properties of measurement instruments. Qual Life Res. 2009;18(8):1115–23. 10.1007/s11136-009-9528-5. 19711195 10.1007/s11136-009-9528-5 PMC2744791

[30] Terwee CB, Bot SD, de Boer MR, van der Windt DA, Knol DL, Dekker J, Bouter LM, de Vet HC. Quality criteria were proposed for measurement properties of health status questionnaires. J Clin Epidemiol. 2007;60(1):34–42. 10.1016/j.jclinepi.2006.03.012. 17161752 10.1016/j.jclinepi.2006.03.012

[31] Terwee CB, Prinsen CAC, Chiarotto A, Westerman MJ, Patrick DL, Alonso J, Bouter LM, de Vet HCW, Mokkink LB. COSMIN methodology for evaluating the content validity of patient-reported outcome measures: a Delphi study. Qual life research: Int J Qual life aspects Treat care rehabilitation. 2018;27(5):1159–70. 10.1007/s11136-018-1829-0. 10.1007/s11136-018-1829-0 PMC5891557 29550964

[32] Cocks K, Cohen D, Wisløff F, Sezer O, Lee S, Hippe E, Gimsing P, Turesson I, Hajek R, Smith A, Graham L, Phillips A, Stead M, Velikova G, Brown J, EORTC Quality of Life Group. An international field study of the reliability and validity of a disease-specific questionnaire module (the QLQ-MY20) in assessing the quality of life of patients with multiple myeloma. Eur J cancer (Oxford England: 1990). 2007;43(11):1670–8. 10.1016/j.ejca.2007.04.022. 10.1016/j.ejca.2007.04.022 17574838

[33] Kontodimopoulos N, Samartzis A, Papadopoulos AA, Niakas D. (2012). Reliability and validity of the Greek QLQ-C30 and QLQ-MY20 for measuring quality of life in patients with multiple myeloma. TheScientificWorldJournal, 2012, 842867. 10.1100/2012/842867 10.1100/2012/842867 PMC3419404 22919356

[34] Espinoza-Zamora JR, Portilla-Espinosa CM, Labardini-Méndez JR, Cervera E, Niesvisky R, Oñate-Ocaña LF. Quality of life in multiple myeloma: clinical validation of the Mexican-Spanish version of the QLQ-MY20 instrument. Ann Hematol. 2015;94(6):1017–24. 10.1007/s00277-014-2290-y. 25563595 10.1007/s00277-014-2290-y

[35] Ahmadzadeh A, Yekaninejad MS, Saffari M, Pakpour AH, Aaronson NK. Reliability and Validity of an Iranian Version of the European Organisation for Research and Treatment of Cancer Quality of Life Questionnaire for Patients with Multiple Myeloma: the EORTC QLQ-MY20. Asian Pac J cancer prevention: APJCP. 2016;17(1):255–9. 10.7314/apjcp.2016.17.1.255. 10.7314/apjcp.2016.17.1.255 26838220

[36] Dachs LR, Gaisán CM, Bustamante G, López SG, García EG, Persona EP, González-Calle V, Auzmendi MS, Pérez JMA, González Montes Y, Ríos Tamayo R, de Miguel Llorente D, Bernal LP, Mayol AS, Caro CC, Grande M, Fernández-Nistal A, Naves A, Miguel EM O. S. Assessment of the psychometric properties of the Spanish version of EORTC QLQ-MY20 and evaluation of health-related quality of Life outcomes in patients with relapsed and/or refractory multiple myeloma in the real-world setting in Spain: results from the CharisMMa study. Leuk Lymphoma. 2023;64(11):1847–56. 10.1080/10428194.2023.2240922. 37539698 10.1080/10428194.2023.2240922

[37] Lorca LA, Sacomori C, Peña C, Barrera C, Salazar M, Leão I, Valladares X, Rojas C. Psychometric properties of the Chilean version of the quality of life questionnaire for multiple myeloma. Revista brasileira de enfermagem. 2024;77(1):e20230100. 10.1590/0034-7167-2023-0100. 38716906 10.1590/0034-7167-2023-0100 PMC11067932

[38] Liu C, Wang L, Wu Y, Jiao Q, Yang B, Jian Y. Psychometric Evaluation of the Chinese Version of the Quality-of-Life Questionnaire for Patients with Multiple Myeloma. Palliat Med Rep. 2025;6(1):98–104. 10.1089/pmr.2024.0086. 40151515 10.1089/pmr.2024.0086 PMC11947649

[39] Osborne TR, Ramsenthaler C, Schey SA, Siegert RJ, Edmonds PM, Higginson IJ. Improving the assessment of quality of life in the clinical care of myeloma patients: the development and validation of the Myeloma Patient Outcome Scale (MyPOS). BMC Cancer. 2015;15:280. 10.1186/s12885-015-1261-6. 25884627 10.1186/s12885-015-1261-6 PMC4404617

[40] Ramsenthaler C, Gao W, Siegert RJ, Schey SA, Edmonds PM, Higginson IJ. Longitudinal validity and reliability of the Myeloma Patient Outcome Scale (MyPOS) was established using traditional, generalizability and Rasch psychometric methods. Qual life research: Int J Qual life aspects Treat care rehabilitation. 2017;26(11):2931–47. 10.1007/s11136-017-1660-z. 10.1007/s11136-017-1660-z PMC5655545 28752440

[41] Gerlach C, Taylor K, Ferner M, Munder M, Weber M, Ramsenthaler C. Challenges in the cultural adaptation of the German Myeloma Patient Outcome Scale (MyPOS): an outcome measure to support routine symptom assessment in myeloma care. BMC Cancer. 2020;20(1):245. 10.1186/s12885-020-06730-7. 32293347 10.1186/s12885-020-06730-7 PMC7092563

[42] Wagner LI, Robinson D Jr, Weiss M, Katz M, Greipp P, Fonseca R, Cella D. Content development for the Functional Assessment of Cancer Therapy-Multiple Myeloma (FACT-MM): use of qualitative and quantitative methods for scale construction. J Pain Symptom Manag. 2012;43(6):1094–104. 10.1016/j.jpainsymman.2011.06.019. 10.1016/j.jpainsymman.2011.06.019 PMC3419469 22575718

[43] Jones D, Vichaya EG, Wang XS, Williams LA, Shah ND, Thomas SK, Johnson VE, Champlin RE, Cleeland CS, Mendoza TR. Validation of the M. D. Anderson Symptom Inventory multiple myeloma module. J Hematol Oncol. 2013;6:13. 10.1186/1756-8722-6-13. 23384030 10.1186/1756-8722-6-13 PMC3598689

[44] Wang T, Chen W, Lin Y, Tang L, Sun J, Ge Y, Mao Y, Liu H. Psychometric properties of the Chinese version of the M.D. Anderson symptom Inventory-Multiple Myeloma Module: a translation and validation study. BMC Cancer. 2025;25(1):640. 10.1186/s12885-025-14054-7. 40200198 10.1186/s12885-025-14054-7 PMC11980299

[45] Gries KS, Fastenau J, Seo C, Potrata B, Iaconangelo C, Serrano D. Development of the Multiple Myeloma Symptom and Impact Questionnaire: A New Patient-Reported Outcome Instrument to Assess Symptom and Impacts in Patients With Multiple Myeloma. Value health: J Int Soc Pharmacoeconomics Outcomes Res. 2021;24(12):1807–19. 10.1016/j.jval.2021.06.010. 10.1016/j.jval.2021.06.010 34838279

[46] Goswami P, Oliva EN, Ionova T, Else R, Kell J, Fielding AK, Jennings DM, Karakantza M, Al-Ismail S, Collins GP, McConnell S, Langton C, Salek S. Development of a Novel Hematological Malignancy Specific Patient-Reported Outcome Measure (HM-PRO): Content Validity. Front Pharmacol. 2020;11:209. 10.3389/fphar.2020.00209. 32210809 10.3389/fphar.2020.00209 PMC7066982

[47] Goswami P, Oliva EN, Ionova T, Else R, Kell J, Fielding AK, Jennings DM, Karakantza M, Al-Ismail S, Collins GP, McConnell S, Langton C, Al-Obaidi MJ, Oblak M, Salek S. Hematological Malignancy Specific Patient-Reported Outcome Measure (HM-PRO): Construct Validity Study. Front Pharmacol. 2020;11:1308. 10.3389/fphar.2020.01308. 33013368 10.3389/fphar.2020.01308 PMC7506039

[48] Salek S, Möller S, Abildgaard N, Rosenberg T, Larsen MT, Asdahl P, Pedersen KK, Lassen MT, Andersen CL, Nielsen LK. Translation, cultural adaptation and validation of the Danish version of the haematological malignancy patient-reported outcome measure (HM-PRO). J patient-reported outcomes. 2025;9(1):43. 10.1186/s41687-025-00869-2. 10.1186/s41687-025-00869-2 PMC12040771 40299237

[49] Ringdal GI, Ringdal K. Testing the EORTC Quality of Life Questionnaire on cancer patients with heterogeneous diagnoses. Qual life research: Int J Qual life aspects Treat care rehabilitation. 1993;2(2):129–40. 10.1007/BF00435732. 10.1007/BF00435732 8518767

[50] Cella DF, Tulsky DS, Gray G, Sarafian B, Linn E, Bonomi A, Silberman M, Yellen SB, Winicour P, Brannon J. The Functional Assessment of Cancer Therapy scale: development and validation of the general measure. J Clin oncology: official J Am Soc Clin Oncol. 1993;11(3):570–9. 10.1200/JCO.1993.11.3.570. 10.1200/JCO.1993.11.3.570 8445433

[51] Portenoy RK, Thaler HT, Kornblith AB, Lepore JM, Friedlander-Klar H, Kiyasu E, Sobel K, Coyle N, Kemeny N, Norton L. The Memorial Symptom Assessment Scale: an instrument for the evaluation of symptom prevalence, characteristics and distress. Eur J cancer (Oxford England: 1990). 1994;30A(9):1326–36. 10.1016/0959-8049(94)90182-1. 10.1016/0959-8049(94)90182-1 7999421

[52] Chang VT, Hwang SS, Feuerman M. Validation of the Edmonton Symptom Assessment Scale. Cancer. 2000;88(9):2164–71. 10.1002/(SICI)1097-0142(20000501)88:9<2164::AID-CNCR24>3.0.CO;2-5. 10813730

[53] Chang VT, Hwang SS, Feuerman M, Kasimis BS, Thaler HT. The memorial symptom assessment scale short form (MSAS-SF). Cancer. 2000;89(5):1162–71. 10.1002/1097-0142(20000901)89:5<1162::AID-CNCR26>3.0.CO;2-Y. 10964347 10.1002/1097-0142(20000901)89:5<1162::aid-cncr26>3.0.co;2-y

[54] Herdman M, Gudex C, Lloyd A, Janssen M, Kind P, Parkin D, Bonsel G, Badia X. Development and preliminary testing of the new five-level version of EQ-5D (EQ-5D-5L). Qual life research: Int J Qual life aspects Treat care rehabilitation. 2011;20(10):1727–36. 10.1007/s11136-011-9903-x. 10.1007/s11136-011-9903-x PMC3220807 21479777

[55] Kim SH, Kim HJ, Lee SI, Jo MW. Comparing the psychometric properties of the EQ-5D-3L and EQ-5D-5L in cancer patients in Korea. Qual life research: Int J Qual life aspects Treat care rehabilitation. 2012;21(6):1065–73. 10.1007/s11136-011-0018-1. 10.1007/s11136-011-0018-1 21947656

[56] Brazier JE, Harper R, Jones NM, O’Cathain A, Thomas KJ, Usherwood T, Westlake L. Validating the SF-36 health survey questionnaire: new outcome measure for primary care. BMJ. 1992;305(6846):160–4. 10.1136/bmj.305.6846.160. 1285753 10.1136/bmj.305.6846.160 PMC1883187

[57] Kristanti MS, Vernooij-Dassen M, Utarini A, Effendy C, Engels Y. Measuring the Burden on Family Caregivers of People With Cancer: Cross-cultural Translation and Psychometric Testing of the Caregiver Reaction Assessment-Indonesian Version. Cancer Nurs. 2021;44(1):37–44. 10.1097/NCC.0000000000000733. 31348026 10.1097/NCC.0000000000000733 PMC7713759

[58] Xu L, Cheng F, Liu C, Jin R, Pan H, Zhang M, Liang Z. Psychometric Properties and Factor Structures of the CERQ, DERS, and RESE Measures: A Bifactor Approach. J Pers Assess. 2021;103(6):797–806. 10.1080/00223891.2021.1887201. 33703970 10.1080/00223891.2021.1887201

[59] Negrini S, Donzelli S, Di Felice F, Zaina F, Caronni A. Construct validity of the Trunk Aesthetic Clinical Evaluation (TRACE) in young people with idiopathic scoliosis. Annals Phys rehabilitation Med. 2020;63(3):216–21. 10.1016/j.rehab.2019.10.008. 10.1016/j.rehab.2019.10.008 31816447

[60] de Vet HC, Terwee CB, Knol DL, Bouter LM. When to use agreement versus reliability measures. J Clin Epidemiol. 2006;59(10):1033–9. 10.1016/j.jclinepi.2005.10.015. 16980142 10.1016/j.jclinepi.2005.10.015

[61] Terwee CB, Mokkink LB, Knol DL, Ostelo RW, Bouter LM, de Vet HC. Rating the methodological quality in systematic reviews of studies on measurement properties: a scoring system for the COSMIN checklist. Qual life research: Int J Qual life aspects Treat care rehabilitation. 2012;21(4):651–7. 10.1007/s11136-011-9960-1. 10.1007/s11136-011-9960-1 PMC3323819 21732199

[62] Calvert, M., King, M., Mercieca-Bebber, R., Aiyegbusi, O., Kyte, D., Slade, A., Chan,A. W., Basch, E., Bell, J., Bennett, A., Bhatnagar, V., Blazeby, J., Bottomley, A.,Brown, J., Brundage, M., Campbell, L., Cappelleri, J. C., Draper, H., Dueck, A. C.,Ells, C., … Wenzel, L. (2021). SPIRIT-PRO Extension explanation and elaboration: guidelines for inclusion of patient-reported outcomes in protocols of clinical trials. BMJ open,11(6), e045105. 10.1136/bmjopen-2020-045105. 10.1136/bmjopen-2020-045105 PMC8246371 34193486

[63] Prinsen CA, Vohra S, Rose MR, Boers M, Tugwell P, Clarke M, Williamson PR, Terwee CB. How to select outcome measurement instruments for outcomes included in a Core Outcome Set - a practical guideline. Trials. 2016;17(1):449. 10.1186/s13063-016-1555-2. 27618914 10.1186/s13063-016-1555-2 PMC5020549

[64] Murphy SL, Berrocal VJ, Poole JL, Khanna D. Reliability, validity, and responsiveness to change of the Patient-Reported Outcomes Measurement Information System self-efficacy for managing chronic conditions measure in systemic sclerosis. J scleroderma Relat disorders. 2022;7(2):110–6. 10.1177/23971983211049846. 10.1177/23971983211049846 PMC9109504 35585951

[65] Barbosa-Silva J, Calixtre LB, Von Piekartz D, Driusso P, Armijo-Olivo S. The minimal important difference of patient-reported outcome measures related to female urinary incontinence: a systematic review. BMC Med Res Methodol. 2024;24(1):60. 10.1186/s12874-024-02188-4. 38459428 10.1186/s12874-024-02188-4 PMC10921720

[66] Silverberg JI, Margolis DJ, Boguniewicz M, Fonacier L, Grayson MH, Ong PY, Fuxench ZC, Simpson EL. Validation of five patient-reported outcomes for atopic dermatitis severity in adults. Br J Dermatol. 2020;182(1):104–11. 10.1111/bjd.18002. 30972740 10.1111/bjd.18002

[67] Cruchinho P, López-Franco MD, Capelas ML, Almeida S, Bennett PM, Miranda da Silva M, Teixeira G, Nunes E, Lucas P, Gaspar F. Translation, Cross-Cultural Adaptation, and Validation of Measurement Instruments: A Practical Guideline for Novice Researchers. J multidisciplinary Healthc. 2024;17:2701–28. 10.2147/JMDH.S419714. & Handovers4SafeCare 10.2147/JMDH.S419714 PMC11151507 38840704

[68] Stratford PW, Riddle DL. Assessing sensitivity to change: choosing the appropriate change coefficient. Health Qual Life Outcomes. 2005;3:23. 10.1186/1477-7525-3-23. 15811176 10.1186/1477-7525-3-23 PMC1084357

[69] Doran CM, Bryant J, Langham E, Bainbridge R, Shakeshaft A, Hobden B, Farnbach S, Freund M. Psychometric properties, and cultural appropriateness, of patient reported outcome measures for use in primary healthcare: a scoping review. Qual life research: Int J Qual life aspects Treat care rehabilitation. 2025;34(8):2137–49. 10.1007/s11136-025-03956-5. 10.1007/s11136-025-03956-5 PMC12274139 40153129

[70] Huang RS, Chen D, Benour A, Cortez R, Mihalache A, Johnny C, Bezjak A, Olson RA, Raman S. Patient-Reported Outcomes as Prognostic Indicators for Overall Survival in Cancer: A Systematic Review and Meta-Analysis. JAMA Oncol. 2025;11(11):1303–12. 10.1001/jamaoncol.2025.3153. 40932728 10.1001/jamaoncol.2025.3153 PMC12426862

[71] Prudêncio DA, Serafim TT, Mateus Lopes M, Maffulli APSR, N., Okubo R. Questionnaires and scales for assessment of ankle function: a systematic review of instruments translated and validated for Brazilian Portuguese. Disabil Rehabil. 2021;43(3):309–16. 10.1080/09638288.2019.1626917. 31184930 10.1080/09638288.2019.1626917

[72] Philipps L, Foster S, Gardiner D, Gath J, Gillman A, Haviland J, Hill E, King D, Manning G, Stiles M, Hall E, Lewis R. Study within a trial of electronic versus paper-based Patient-Reported oUtcomes CollEction (SPRUCE): study protocol for a partially randomised patient preference study. BMJ open. 2023;13(9):e073817. 10.1136/bmjopen-2023-073817. 37734892 10.1136/bmjopen-2023-073817 PMC10514621

[73] Vanier A, Leroy M, Hardouin JB. Toward a rigorous assessment of the statistical performances of methods to estimate the Minimal Important Difference of Patient-Reported Outcomes: A protocol for a large-scale simulation study. Methods (San Diego Calif). 2022;204:396–409. 10.1016/j.ymeth.2022.02.006. 35202798 10.1016/j.ymeth.2022.02.006

