# Supplementary material for: Psychometric properties of patient-reported outcome measures for health-related quality of life in multiple myeloma: a systematic review based on COSMIN guidelines
Source: BMC Cancer. 2026 Jun 15;26:1014. doi: 10.1186/s12885-026-16341-3 (PMC13501831; doi:10.1186/s12885-026-16341-3)
Supplement: Supplementary file 1 [file 12885_2026_16341_MOESM1_ESM.docx]

Supplementary tables and information

**Supplementary Table S1. Predefined criteria used to rate measurement properties.**

| **Measurement property** | **Sufficient rating** | **Predefined COSMIN criterion used in this review** |
| --- | --- | --- |
| Content validity | + | Items relevant for construct, target population and context; no key concepts missing; items/response options/instructions understood as intended |
| Structural validity | + | CTT: loadings ≥0.30, ≤10% cross-loadings, explained variance ≥50% and structure consistent with theory; CFA: CFI/TLI >0.95 or RMSEA <0.06 or SRMR <0.08; IRT/Rasch criteria met |
| Internal consistency | + | At least low evidence for sufficient unidimensionality and Cronbach’s alpha ≥0.70 |
| Cross-cultural validity/measurement invariance | + | No important group differences in MGCFA or no important DIF |
| Reliability | + | ICC, weighted kappa or Pearson/Spearman correlation ≥0.70 |
| Measurement error | + | SDC or LoA smaller than MIC |
| Criterion validity | + | Correlation with gold standard ≥0.70 or AUC ≥0.70, when a true gold standard exists |
| Hypothesis testing for construct validity | + | ≥75% of results in accordance with predefined hypotheses |
| Responsiveness | + | ≥75% of results in accordance with predefined hypotheses or AUC ≥0.70 |

*Note. These criteria should also be summarized in the Methods section because they define how COSMIN ratings were assigned.*

**Supplementary Table S2. Feasibility and interpretability considerations for the included PROMs.**

| **PROM** | **Length** | **Completion time** | **Scoring burden** | **Copyright/cost/approval** | **Interpretability notes** |
| --- | --- | --- | --- | --- | --- |
| QLQ-MY20 | 20 items + QLQ-C30 core if EORTC system used | Approximately 12 minutes reported for QLQ-MY20 in original field study | Low to moderate; requires EORTC scoring transformation | EORTC permission/manual required | Some ceiling effects led to removal of social support items in development; MIC not consistently available in included studies |
| MyPOS | 27 scored items/30 total | Mean completion time 7 min 19 s reported in development study | Low; designed for clinical care | King's/POS licensing conditions should be checked. | MID reported in longitudinal study (+2.5 improvement; –4.5 deterioration); floor effects noted |
| FACT-MM | 14-item MM module plus FACT-G | NR in included evidence | Moderate; FACIT scoring rules | FACIT permission/licence usually required | Interpretability evidence limited in included evidence |
| MDASI-MM | 26 items | Chinese version reported 5–7 minutes | Low; 0–10 ratings easy to score | MD Anderson inventory permission may be required | Good for symptom tracking; MIC/measurement error limited |
| MySIm-Q | 17 items | NR | Low to moderate | Developer/sponsor permission may be needed | Content validity supported; interpretability/MIC evidence lacking |
| QLICP-MM | 43 items | NR | Moderate; domain transformation and MCID procedures reported | NR | MCID work reported; COSMIN measurement error still not established |
| HM-PRO | 42 items | NR | Moderate; two-part scoring | Permission may be required | Broader hematological malignancy use; MM-specific interpretability limited |
| QLQ-C30 | 30 items | Generally brief; NR in included study | Moderate; EORTC transformed scoring | EORTC permission/manual required | Legacy interpretation aids widely available, but not re-synthesized here |
| FACT-G | 28 items in original validation | Generally brief; NR in included study | Moderate; FACIT scoring | FACIT permission/licence usually required | Legacy interpretation aids exist; MM-specific thresholds not identified |
| MSAS | 32 symptoms | NR; longer than ESAS/MSAS-SF | Moderate; multiple symptom dimensions | NR | Subscale scores available; MIC not identified |
| ESAS | 9 core symptoms + optional item | Very brief | Low; item-level 0–10 scoring | Generally freely used in clinical settings; verify local rules | Cut-off values may vary by symptom; 30-mm cutoff not uniform |
| MSAS-SF | 32 symptoms but short form | Shorter than MSAS; NR | Low to moderate | NR | Subscales PHYS/PSYCH/GDI available; retest varies by interval |
| EQ-5D-5L | 5 dimensions + EQ VAS | Very brief | Low for descriptive profile; utility scoring requires value set | EuroQol registration/licence required | Utility interpretation depends on country-specific value sets |
| SF-36 | 36 items | Approximately 5 minutes reported in validation study | Moderate; domain scoring algorithms | RAND/SF licensing depends on version | Generic norms and interpretation available; MM-specific MIC not synthesized |

*Note. NR = not reported; MIC = minimal important change. Feasibility is not a measurement property but is important for clinical and research selection of PROMs.*

## Abbreviations used in tables

A = adequate; COSMIN = COnsensus-based Standards for the selection of health Measurement INstruments; CFA = confirmatory factor analysis; CTT = classical test theory; HRQoL = health-related quality of life; ICC = intraclass correlation coefficient; I = inadequate; IRT = item response theory; MIC = minimal important change; MGCFA = multi-group confirmatory factor analysis; MI = measurement invariance; MM = multiple myeloma; NR = not reported; PROM = patient-reported outcome measure; RoB = risk of bias; SDC = smallest detectable change; V = very good.

**S3 Supplementary Information: Literature search strategy**

1. PubMed, August 26, 2025

#1 ("Multiple Myeloma"[Mesh] OR "multiple myeloma"[Title/Abstract] OR "relapsed myeloma"[Title/Abstract] OR "refractory myeloma"[Title/Abstract] OR "relapsed/refractory myeloma"[Title/Abstract] OR "relapsed/refractory multiple myeloma"[Title/Abstract])

#2 ("Quality of Health Care"[Mesh] OR "Quality of Life"[Mesh] OR "Patient Reported Outcome Measures"[Mesh] OR "health-related quality of life"[Title/Abstract] OR "quality of life"[Title/Abstract] OR "patient-reported outcome measure"[Title/Abstract] OR "patient reported outcome"[Title/Abstract])

#3 ("psychometr*"[Title/Abstract] OR "valid*"[Title/Abstract] OR "reliab*"[Title/Abstract] OR "coefficient"[Title/Abstract] OR "internal consistency"[Title/Abstract] OR "Cronbach"[Title/Abstract] OR "alpha"[Title/Abstract] OR "item"[Title/Abstract] OR "correlation"[Title/Abstract] OR "test-retest"[Title/Abstract] OR "factor analysis"[Title/Abstract] OR "factor structure"[Title/Abstract] OR "subscale"[Title/Abstract] OR "measurement invariance"[Title/Abstract] OR "cross-cultural"[Title/Abstract] OR "instrumentation"[MeSH Subheading] OR "methods"[MeSH Subheading] OR "Psychometrics"[MeSH Terms] OR "Validation Study"[Publication Type] OR "Comparative Study"[Publication Type])

#4 #1 AND #2 AND #3 (781 results)

2. Embase, August 26, 2025

#1 'multiple myeloma' OR 'refractory myeloma' OR 'relapsed myeloma' OR 'relapse myeloma' OR 'relapsed/refractory myeloma' OR 'relapsed/refractory multiple myeloma'

#2 'psychometry' OR 'psychometric properties' OR 'quality of life' OR 'quality of life assessment' OR 'quality of life questionnaire' OR 'patient-reported outcome' OR 'patient reported outcome measure' OR 'outcome assessment'

#3 'validity' OR 'reliability' OR 'internal consistency' OR 'cronbach alpha coefficient' OR 'factor analysis' OR 'test-retest' OR 'measurement invariance' OR 'cross-cultural adaptation'

#4 #1 AND #2 AND #3 (1046 results)

3. Cochrane Library, August 26, 2025

#1 MeSH descriptor: [Multiple Myeloma] explode all trees

#2 MeSH descriptor: [Patient Reported Outcome Measures] explode all trees OR MeSH descriptor: [Quality of Life] explode all trees

#3 MeSH descriptor: [Psychometrics] explode all trees OR MeSH descriptor: [Validation Studies as Topic] explode all trees

#4 #1 AND #2 AND #3 (545 results)

4. CINAHL, August 26, 2025

S1 TI ("multiple myeloma" OR "refractory myeloma" OR "relapsed myeloma" OR "relapse myeloma")

S2 TI ("Quality of Health Care" OR "health-related quality of life" OR "quality of life") OR AB ("Patient Reported Outcome Measures" OR "patient-reported outcome")

S3 AB (instrumentation OR measure OR psychometrics OR psychometr* OR measurement) AND AB (alpha OR alphas OR correlation* OR selection* OR reduction* OR test-retest OR retest OR reliab* OR reproducible* OR discriminant analysis OR valid* OR homogeneity OR cronbach* OR internal consistency OR item OR coefficient)

S4 S1 AND S2 AND S3 (204 results)

5. SinoMed, August 26, 2025

("Multiple Myeloma"[Title] OR "Relapsed Myeloma"[Title] OR "Refractory Myeloma"[Title]) AND ("Quality of Life"[Title] OR "Health-Related Quality of Life"[Title] OR "Survival Quality"[Title]) AND ("Questionnaire"[Title] OR "Scale"[Title] OR "Instrument"[Title] OR "Validity"[Title] OR "Reliability"[Title] OR "Test"[Title] OR "Validation"[Title] OR "Cross-Cultural"[Title] OR "Cross-Cultural Adaptation"[Title] OR "Measurement Properties"[Title]) (95 results)

6. Web of Science, August 26, 2025

#1 TS=("multiple myeloma" OR "refractory myeloma" OR "relapsed myeloma" OR "relapse myeloma")

#2 TS=("Quality of Health Care" OR "quality of life" OR "health-related quality of life" OR "Patient Reported Outcome Measures" OR "patient-reported outcome")

#3 #1 AND #2

#4 TS=(Instrument OR Scale OR Questionnaire OR Measure OR Index OR Test OR Checklist OR Tool OR Assess* OR Screen*)

#5 TI=(Valid* OR Reliable OR Psychometric* OR "Factor Analy*" OR "Internal Consistency" OR Reproducibility OR "cross-cultural equivalence" OR "cross-cultural adaptation")

#6 #3 AND #4 AND #5 (679 results)

7. Wanfang Database, August 26, 2025

(Topic: Multiple Myeloma OR Relapsed Myeloma OR Refractory Myeloma) AND (Title/Keywords: Quality of Life OR Health-Related Quality of Life OR Survival Quality) AND (Title: Questionnaire OR Scale OR Instrument OR Validity OR Reliability OR Test OR Validation OR Cross-Cultural OR Cross-Cultural Adaptation OR Measurement Properties) (223 results)

8. China National Knowledge Infrastructure (CNKI), August 26, 2025

(Topic/Abstract/Keywords: Multiple Myeloma OR Relapsed Myeloma OR Refractory Myeloma) AND (Topic/Abstract/Keywords: Quality of Life OR Health-Related Quality of Life OR Survival Quality) AND (Topic/Abstract/Keywords: Questionnaire OR Scale OR Instrument OR Validity OR Reliability OR Test OR Validation OR Cross-Cultural OR Cross-Cultural Adaptation OR Measurement Properties) (60 results)
